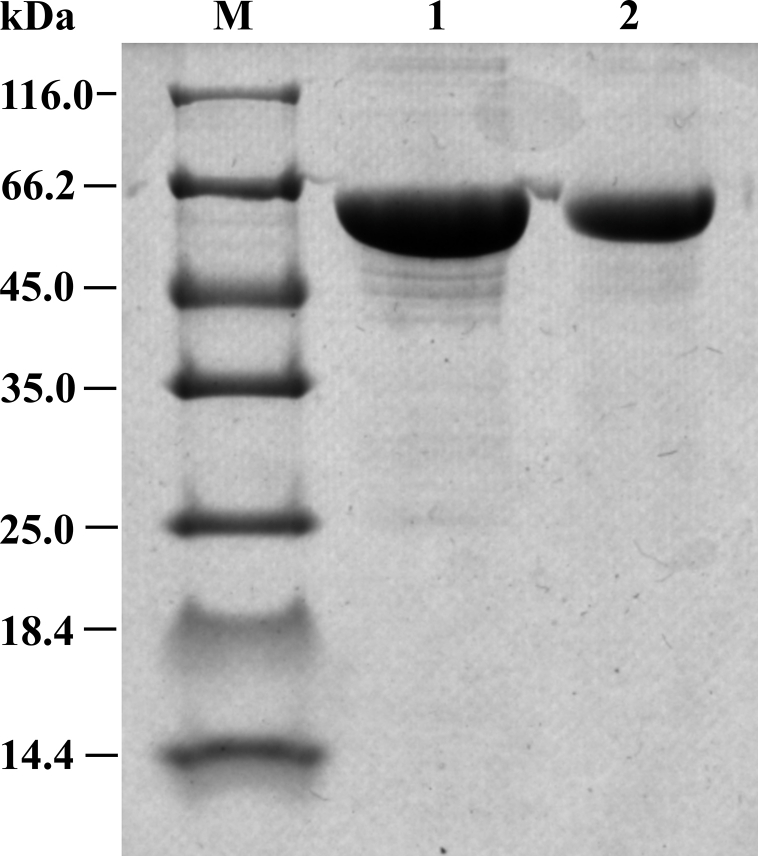# Improvement of aflatoxin B_1_ degradation ability by *Bacillus licheniformis* CotA-laccase Q441A mutant

**DOI:** 10.1016/j.heliyon.2023.e22388

**Published:** 2023-11-13

**Authors:** Yanrong Liu, Yongpeng Guo, Limeng Liu, Yu Tang, Yanan Wang, Qiugang Ma, Lihong Zhao

**Affiliations:** a State Key Laboratory of Animal Nutrition and Feeding, Poultry Nutrition and Feed Technology Innovation Team, College of Animal Science and Technology, China Agricultural University, Beijing, 100193, PR China; b College of Animal Science and Technology, Henan Agricultural University, Zhengzhou, 450046, PR China

**Keywords:** CotA-laccase, *Bacillus licheniformis*, Site-directed mutagenesis, Aflatoxin B_1_

## Abstract

Aflatoxin B_1_ (AFB_1_) contamination seriously threatens nutritional safety and common health. Bacterial CotA-laccases have great potential to degrade AFB_1_ without redox mediators. However, CotA-laccases are limited because of the low catalytic activity as the spore-bound nature. The AFB_1_ degradation ability of CotA-laccase from *Bacillus licheniformis* ANSB821 has been reported by a previous study in our laboratory. In this study, a Q441A mutant was constructed to enhance the activity of CotA-laccase to degrade AFB_1_. After the site-directed mutation, the mutant Q441A showed a 1.73-fold higher catalytic efficiency (*k*_cat_/K_m_) towards AFB_1_ than the wild-type CotA-laccase did. The degradation rate of AFB_1_ by Q441A mutant was higher than that by wild-type CotA-laccase in the pH range from 5.0 to 9.0. In addition, the thermostability was improved after mutation. Based on the structure analysis of CotA-laccase, the higher catalytic efficiency of Q441A for AFB_1_ may be due to the smaller steric hindrance of Ala441 than Gln441. This is the first research to enhance the degradation efficiency of AFB_1_ by CotA-laccase with site-directed mutagenesis. In summary, the mutant Q441A will be a suitable candidate for highly effective detoxification of AFB_1_ in the future.

## 1 Introduction

Mycotoxins are secondary metabolites produced by molds [1]. Nearly 25 % of the world's food supply is contaminated with mycotoxins each year [2]. The most common mycotoxins in food and feed are aflatoxins [3]. The four main natural aflatoxins are aflatoxin B_1_ (AFB_1_), aflatoxin B_2_, aflatoxin G_1_ and aflatoxin G_2_ [4]. Among these four kinds of aflatoxins, AFB_1_ receives particular attention because of the severe carcinogenicity and immunotoxicity to humans and animals [5]. Different approaches have been used to remove AFB_1_ from grains, such as ammoniation, irradiation, adsorption, ozonation and peroxidation [6]. However, these methods are expensive and cause adverse effects on nutritional value of cereals [6]. Because of the depressed performance of traditional physical and chemical approaches to remove AFB_1_, biological methods have shown great potential in AFB_1_ degradation [7]. At present, there are many reports about the enzymatic detoxification of AFB_1_. Among these enzymes, laccase has attracted much attention for its good detoxification effect [8].

Laccases are enzymes that can catalyze various phenolic compounds, and reduce molecular oxygen to water to oxidize aromatic compounds [9]. They are widely distributed among plants, insects, fungi and bacteria [10]. In plants, laccases are implicated in cell wall lignification [11]. In insects, laccases participate in lignin degradation and cuticle sclerotization [12]. In fungi, laccases are related to plant pathogenesis and lignin degradation [13]. In bacteria, laccases are involved in melanin production and copper detoxification [14].

Laccases have been considered to be biological green tools to remove environmental pollutants [15]. A number of laccases, such as fungal and bacterial laccases, have been demonstrated to have the ability to degrade AFB_1_. The laccases from *Trametes versicolor* could directly oxidize AFB_1_, and the mutagenicity and prooxidative abilities of the degradation products were reduced compared to AFB_1_ [16]. Song and coworkers reported that *Pleurotus pulmonarius* laccase 2 was able to detoxify 99.82 % of AFB_1_ in the presence of acetosyringone [17]. However, fungal laccases are usually unstable under high temperature and alkaline condition, thus limiting their practical applications in aflatoxin detoxification in food and feed processing as several processing steps (such as maize gelatinization processing, maize puffing, and the processing of pellet feed) occur at elevated temperatures [15]. Compared with fungal laccases, bacterial laccases offer the advantage of better alkali and high-temperature resistance, so that have broader application prospects in the biotechnology [18].

In recent years, the detoxification ability of various enzymes to mycotoxins has been improved mainly by molecular modification. Structure-guided mutagenesis has been used to improve the degradability of zearalenone by different zearalenone hydrolases [19,20]. Furthermore, the catalytic performance of *Trametes versicolor* AFB_1_-degrading enzyme was improved by mutating Gln232 to Met232 [21]. Our previous study has reported a CotA-laccase from *Bacillus licheniformis* ANSB821 (accession no. MN075270). This CotA-laccase can oxidize AFB_1_ in the absence of redox mediators and exhibits excellent thermostability [8]. However, the ability of CotA-laccase to degrade AFB_1_ under acidic or neutral conditions is not as high as that under alkaline conditions, which limits the detoxification of AFB_1_ by CotA-laccase in the gastrointestinal tract of animals. To improve the AFB_1_ degradation efficiency of CotA-laccase and widen the potential application in degrading AFB_1_ in food and feed, a combination of structure-based methods and site-directed mutagenesis was used in the present study. Based on this, the mutant enzyme was constructed and compared with the wild-type CotA-laccase according to the kinetic parameters, optimal pH and temperature, as well as detoxification ability to AFB_1_.

## 2 Methods and materials

### 2.1 Materials

2,2′-Azino-bis (3-ethylbenzothiazoline-6-sulfonic acid) (ABTS) was purchased from Sigma-Aldrich (St. Louis, MO, USA). Aflatoxin B_1_ (AFB_1_) was purchased from Pribolab (Qingdao, China). Isopropyl-β-d-thiogalactopyranoside (IPTG) was obtained from Biotopped (Beijing, China). Ampicillin was from Solarbio (Beijing, China). The plasmid extraction kit and fast mutagenesis system were obtained from Tiangen (Beijing, China). All chemical reagents were standard reagent grade.

### 2.2 Plasmid, strains, and media

The CotA-laccase gene of *B. licheniformis* ANSB821 stored in our laboratory was expressed into pET31b provided by Invitrogen (Carlsbad, CA). *Escherichia coli* strain DH5α and BL21 cells were obtained from TransGen Biotech (Beijing, China). All strains were cultivated with Luria-Bertani (LB) medium.

### 2.3 Bioinformatics analysis

All protein sequences were searched from the NCBI (National Center for Biotechnology Information, https://www.ncbi.nlm.nih.gov). Multiple sequence alignment was performed with Clustal Omega (http://www.ebi.ac.uk/Tools/msa/clustalo/). Homology modeling was performed with SWISS-MODEL Server (http://swissmodel.expasy.org/). CotA-laccase protein from *B. subtilis* (PDB code: 1GSK) that showed 66.14 % identity to *B. licheniformis* laccase was used as the template. The structures of enzymes were analyzed with PyMol viewer. The substrate molecules were docked into the enzyme structure with Software AutoDockTools 1.5.6.

### 2.4 Construction of mutant

The mutant was constructed by Quik-Change site specific mutagenesis. PCR mixture contained the synthetic primers as Table S1, fastalteration buffer, fastalteration DNA polymerase, RNase-free ddH_2_O and plasmid pET31b which contained the wild-type CotA-laccase gene. The PCR program consists of pre-heating the reaction mixture at 95°C for 2 min, followed by 18 cycles of denaturation at 94°C (20 s), annealing at 55°C (10 s) and extension at 68°C for 2.5 min with a final extension at 68°C for 5 min. The PCR product was incubated with *Dpn*Ⅰ restriction enzyme at 37°C for 1 h, and the template plasmid was removed. Finally, the digested product was transformed into *E. coli* DH5α and sequenced to confirm the mutant. Confirmed gene was then transformed into *E. coli* BL21 by heat shock transformation method.

### 2.5 Expression and purification

Expression of CotA-laccase and its mutant was based on our previous work [8]. The recombinant strains were cultured at 37°C in LB medium containing 100 μg mL^−1^ ampicillin. 0.1 mM IPTG and 2 mM CuSO_4_ were added to induce the gene expression at an OD_600_ of 0.6 in LB medium. Temperature was reduced to 16°C in a shaking incubator (120 rpm) and cells were incubated for 20 h. Afterwards, cells were harvested by centrifugation (15 min, 8000×*g*, 4°C) and the pellets were resuspended in binding buffer (50 mM sodium phosphate, 500 mM NaCl, 5 mM imidazole, pH 7.4). The cells were disrupted by sonification on ice and the cell debris was removed by centrifugation (10 min, 12,000×*g*, 4°C).

The proteins in supernatant were then purified by Ni-NTA affinity chromatography column (Qiagen, Chatsworth, CA, USA) as described previously [8]. The His-tags of both wild-type CotA-laccase and Q441A were not removed in this study. Purity of the proteins was confirmed by 12 % sodium dodecyl sulfate polyacrylamide gel electrophoresis (SDS-PAGE). Then the collected proteins were concentrated by ultrafiltration (molecular weight cutoff of 30 kDa) and quantified using a Bradford Protein Assay Kit (TransGen, China).

### 2.6 Enzyme activity assay

The kinetic parameters of purified laccases were measured at 37°C with ABTS as substrate. The reaction mixture (3 mL total volume) contained 2.0 μg mL^−1^ CotA-laccase, 100 mM sodium citrate buffer (pH 4.5), and various amounts of ABTS (5–1000 μM). The ultraviolet absorbance of ABTS was 420 nm (ɛ = 36,000 M^−1^cm^−1^) [22]. One unit of enzyme activity was defined as 1 μmol of substrate oxidized by how many amounts of enzyme per minute. Kinetic parameters were fitted by nonlinear Michaelis-Menten plots with Graphpad Prism 8.0 (SanDiego, CA, USA). All assays were performed at least in triplicate.

### 2.7 Optimum pH and pH stability

The optimum pH of wild-type CotA-laccase and the mutant was measured with ABTS as substrate. The reaction mixture (3 mL total volume) contained 2.0 μg mL^−1^ CotA-laccase, 100 mM sodium citrate buffer (pH 4.5), and 1 mM ABTS. The reaction was run at 37°C for 30 s, and the enzyme activities were spectrophotometrically determined at 420 nm. The pH stability of wild-type CotA-laccase and the mutant was assayed by overnight incubation at 4°C and different pH values (pH 3.0–12.0), and then residual activity was measured. The remaining enzyme activity was determined at 37°C and pH 4.5 with ABTS as substrate. All assays were done at least in triplicate.

### 2.8 Optimum temperature and thermal stability

The optimum temperature of wild-type CotA-laccase and the mutant was measured with ABTS as substrate. The reaction mixture (3 mL total volume) contained 2.0 μg mL^−1^ CotA-laccase, 100 mM sodium citrate buffer (pH 4.5), and 1 mM ABTS. The reaction solutions were maintained at different temperatures (30-100°C) for 30 s, and the enzyme activities were spectrophotometrically determined at 420 nm. The thermal stability was determined by incubating enzymes in 100 mM sodium phosphate buffer (pH 7.5) at 50, 70 and 90°C, and then the residual activity was measured. The remaining enzyme activity was determined at 37°C and pH 4.5 with ABTS as substrate. All measurements were performed at least in triplicate.

### 2.9 Aflatoxin oxidase properties

All AFB_1_ oxidation assays were performed in the 2 mL centrifuge tubes with a reaction volume of 500 μL, containing 20 μg mL^−1^ of CotA and 2.0 μg mL^−1^ of AFB_1_. The effect of pH on AFB_1_ oxidation by wild-type CotA-laccase and the mutant was determined at pH 5.0–9.0 and 37°C for 12 h. The effect of temperature on AFB_1_ oxidation was determined in the range of 30–80°C and pH 8.0 for 30 min. The effect of metal ions on CotA-mediated oxidation of AFB_1_ was determined. The wild-type CotA-laccase and the mutant (100 μg mL^−1^) were pre-incubated with 10 mM metal ions at 37°C and pH 8.0 for 10 min. Then AFB_1_ was added and the mixture reacted at 37°C for 30 min. The control was prepared in the absence of the enzymes. The reaction was terminated by adding 500 μL HPLC-grade methanol. The assays were measured at 37°C and pH 8.0 using different concentrations (1–100 μg mL^−1^) of AFB_1_ as substrates when calculated the kinetic parameters [8]. The initial reaction rate was measured by monitoring the reduction of AFB_1_ every 6 min intervals for a total of 30 min. Kinetic parameters were calculated by nonlinear Michaelis-Menten plots with Graphpad Prism 8.0 (SanDiego, CA, USA). All assays were performed at least in triplicate. The degradation rate of AFB_1_ was calculated as the following equation: *D*_*R*_ = (1 - *C*_*T*_/*C*_*C*_) × 100 %, where *D*_*R*_ was the degradation rate; *C*_*T*_ and *C*_*C*_ were the concentration of AFB_1_ in the experimental group and the control group, respectively.

### 2.10 Statistical analysis

Data analysis was performed by GraphPad Prism 8.3 software (GraphPad Software, USA). Data were shown as the averages ± standard deviation of three parallel experiments. Two-tailed unpaired Student's *t*-test was used for comparison between two groups and one-way ANOVA followed Tukey's *t*-test was used for comparison between multiple groups. *P* < 0.05 was statistically significant.

## 3 Results

### 3.1 Structural model of B. licheniformis CotA-laccase

The combination technique of structure-based approaches and site-directed mutagenesis was used for purpose of enhancing the catalytic activity of CotA-laccase. In this experiment, the 3D structure of *B. licheniformis* ANSB821 CotA-laccase was obtained by homology modeling. Hence, it has been assumed that the generated model could stand for the structure of CotA-laccase from *B. licheniformis*. Laccases were members of the multicopper oxidase family with three copper centers [23]. Laccases were reported to be able to utilize a wide range of substrates [24]. There were three cupredoxin-like domains in structure of CotA-laccase, and four copper atoms existed in the form of type-1, type-2 and type-3 copper centers [25]. The catalytic mechanism of laccase includes oxidation of the substrate on type-1 Cu site and reduction of O_2_ to H_2_O on type-2/3 Cu cluster [26]. Shown here, Gln441 of *B. licheniformis* CotA-laccase was located in the α-helix fragment of domain 3, which was the entrance of substrate-binding pocket (Fig. 1). It was surmised that the different conformation of this site may affect the activity of laccase.

**Fig. 1:**
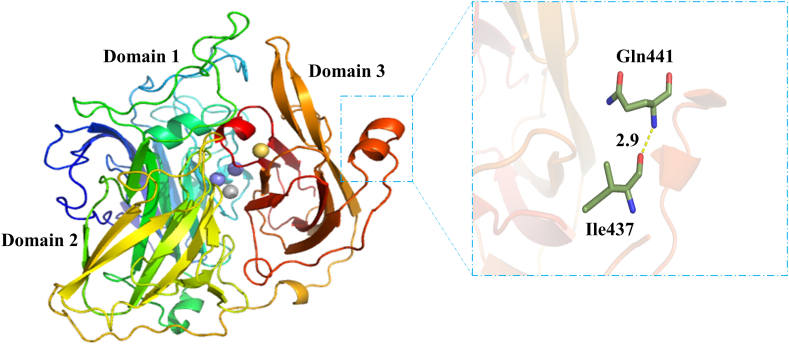
The modeled 3D structure of *B. licheniformis* CotA-laccase. This molecular structure is comprised of three domains, and four copper atoms are classified into three types which are marked with different colors. The position of Gln441 and its hydrogen bonding interaction with Ile437 are highlighted as the close-up view.

### 3.2 Mutation design, cloning and expression

Site-directed mutagenesis has so far been an important method to improve the enzyme activity. In a previous study, the mutant Q442A of *B. pumilus* W3 screened from the saturation mutagenesis library showed 2.45-fold higher catalytic efficiency on ABTS than the wild-type CotA-laccase [27]. Moreover, the Reactive Black 5 decolorization activity was improved by site-saturation mutagenesis. The potential mutation site of CotA-laccase, Gln442, plays an important role in changing the affinity of substrate [28]. According to the sequence alignment of amino acid residues of *B. licheniformis* ANSB821 and *B. pumilus* W3 CotA-laccase, Gln441 in *B. licheniformis* laccase was equivalent to Gln442 in *B. pumilus* laccase (Fig. S1). Therefore, it was hypothesized that the catalytic activity of the CotA-laccase in our present study for ABTS and the degradation ratio for AFB_1_ could be improved by replacing Gln441 with Ala441. In order to study the possible effect of single amino acid replacement on the catalytic activity of *B. licheniformis* CotA-laccase, mutation Q441A was successfully introduced into the CotA-laccase gene of *B. licheniformis*. In our previous study, plasmid containing the gene of CotA-laccase of *B. licheniformis* ANSB821 has been introduced into *E. coli*. In this study, both the wild-type CotA-laccase and Q441A mutant were cloned and expressed in *E. coli*.

### 3.3 Purification and characterization of wild-type CotA-laccase and Q441A mutant

The recombinant wild-type CotA-laccase and its variant were purified by Ni-NTA affinity chromatography column as described previously [8]. The two purified enzymes molecular weight were ∼60 kDa (Fig. S2). To study the enzymatic properties of these two enzymes, ABTS was used as the substrate. The *k*_cat_ and K_m_ values of wild-type CotA-laccase and Q441A mutant to ABTS were listed in Table 1. The *k*_cat_/K_m_ value of Q441A was determined to be 392.07 s^−1^ mM^−1^, which was about 2.02-folds higher than the 194.09 s^−1^ mM^−1^ of wild-type laccase.

**Table 1:** Kinetic parameters for the wild-type CotA and Q441A using ABTS as substrate.

Laccase	K_m_ (mM)^a^	*k*_cat_ (s^−1^)	*k*_cat_/K_m_ (s^−1^ mM^−1^)
WT	0.079 ± 0.005	15.36 ± 2.41	194.09
Q441A	0.052 ± 0.003	20.48 ± 2.18	392.07

a Laccase activity was calculated at 37°C with 100 mM sodium citrate buffer (pH 4.5). ABTS was chosen as substrate. K_m_ and *k*_cat_ were determined by Michaelis-Menten equation.

### 3.4 Effect of pH on enzyme activity and stability

**Fig. 2:**
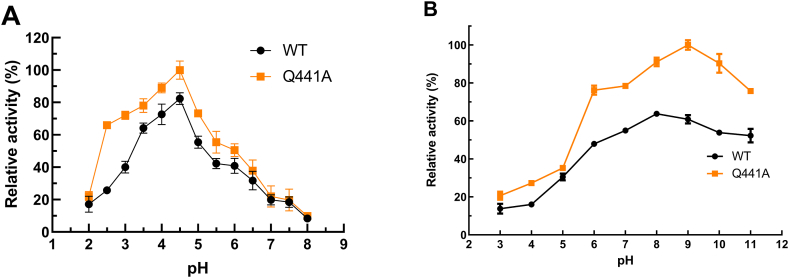
Effect of pH on the activity **(A)** and stability **(B)** of the purified wild-type CotA-laccase and Q441A at 37°C using ABTS as substrate. **(A)** The test on enzyme activity was conducted at pH 2.0–8.0, whereas **(B)** the test on stability involved the incubation of the purified CotA for overnight at 4°C and pH 3.0–12.0 before measurement of residual activity.

In this study, the optimal pH value of both wild-type CotA-laccase and Q441A were 4.5 with ABTS as substrate (Fig. 2A). Moreover, the catalytic activity at 2.5 and 3.0 were higher for Q441A compared with the wild-type CotA-laccase. Stability studies of pH indicated that the wild-type CotA-laccase and Q441A were especially stable in a neutral and alkaline environment (Fig. 2B). The wild-type CotA-laccase was most stable at pH 8.0, and Q441A was most stable at pH 9.0. The wild-type CotA-laccase maintained 22 % residual activity after overnight incubation at pH 3.0 compared to pH 8.0. Similarly, Q441A maintained 21 % residual activity after overnight incubation at pH 3.0 compared to pH 9.0. The pH stability of Q441A had no distinct difference with that of the wild-type at acidic pHs. However, the activity of Q441A after overnight incubation at pH 9.0 was about 1.57-folds higher than the activity of wild-type CotA-laccase after overnight incubation at pH 8.0. Therefore, although the stability of Q441A and wild-type CotA-laccase was similar, the residual activity of Q441A was higher than that of wild-type CotA-laccase after overnight incubation at each pH.

### 3.5 Effect of temperature on enzyme activity and stability

**Fig. 3:**
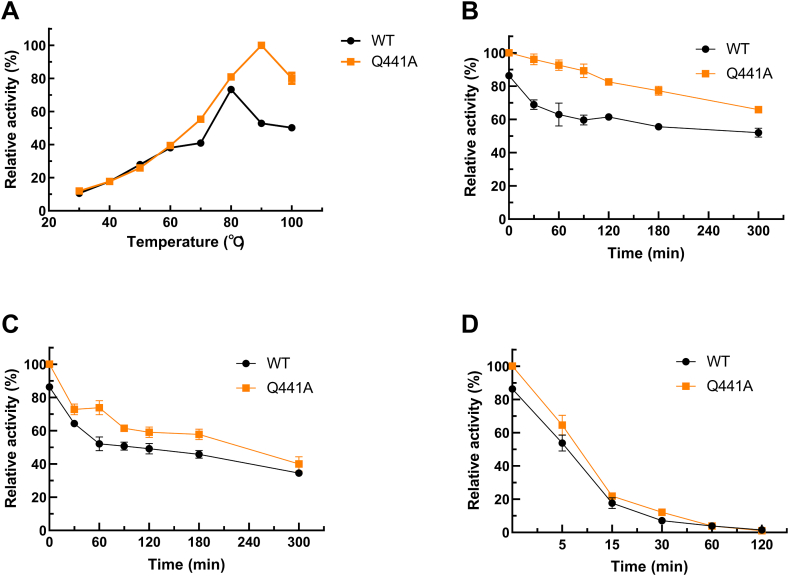
Effect of temperature on the activity **(A)** and stability **(B**–**D)** of the purified wild-type CotA-laccase and Q441A using ABTS as substrate at pH 4.5. **(A)** Laccase activity was measured at different temperatures (30-100°C). The thermostability of the wild-type CotA-laccase and Q441A were measured after incubation at different temperatures 50°C **(B)**, 70°C **(C)**, and 90°C **(D)**, respectively.

The wild-type CotA-laccase had the optimal reaction temperature at 80°C with ABTS as substrate. The Q441A held the optimal temperature at 90°C (Fig. 3A). As for the thermostability of the two CotA-laccases, it demonstrated a higher thermostability at 50°C and 70°C for Q441A (Fig. 3B–D). Furthermore, both the two enzymes were stable at 50°C for maintaining more than 60 % activity after incubated for 5 h.

### 3.6 Catalytic efficiency of aflatoxin B_1_ by wild-type CotA-laccase and Q441A mutant

**Fig. 4:**
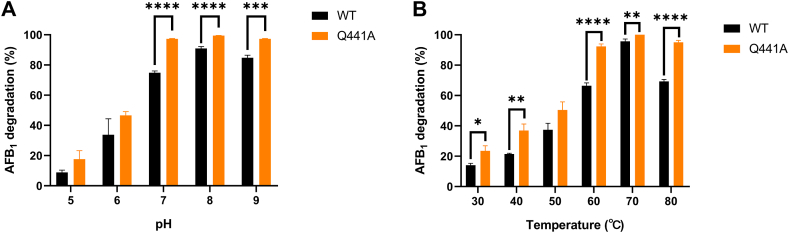
Effect of pH **(A)** and temperature **(B)** on the activity of the purified wild-type CotA-laccase and Q441A using AFB_1_ as substrate.

The application of CotA-laccase from *B. licheniformis* ANSB821 in AFB_1_ detoxification has been reported [8]. In this study, the catalytic efficiency of AFB_1_ by purified wild-type CotA-laccase and Q441A were compared. As shown in Table 2, the purified Q441A mutant exhibited higher *k*_cat_/K_m_ value on AFB_1_ than the wild-type CotA-laccase (1.73-fold). This was attributed to the decrease in K_m_ and the slightly increase in *k*_cat_ for the Q441A mutant. The effects of pH and temperature on the oxidation activity of wild-type CotA-laccase and Q441A with AFB_1_ as substrate were shown in Fig. 4. Compared with the wild-type CotA-laccase, the degradation of AFB_1_ by Q441A increased in the pH range from 5.0 to 9.0. The AFB_1_ degradation ratio by Q441A was more than 97 % in the pH range from 7.0 to 9.0, whereas, the maximal AFB_1_ degradation rate by wild-type CotA-laccase reached up to 91 % only at pH 8.0. The results showed that compared with the wild-type laccase, the AFB_1_ degradation rate of Q441A was improved under acid, neutral and alkaline conditions. The optimal temperature for Q441A to degrade AFB_1_ was 70°C, which was the same as wild-type laccase, and the degradation of AFB_1_ by Q441A increased in the range of 30–80°C. In addition, the AFB_1_ degradation rate of Q441A was higher than that of the wild-type laccase significantly at 30, 40, 60, 70, and 80°C. The effect of metal ions (10 mM) on the activity of wild-type CotA-laccase and Q441A towards AFB_1_ was shown in Table 3. Among the seven metal ions, Cu^2+^, Mg^2+^ and Na^+^ had little effect on the activity of laccases towards AFB_1_. The significant inhibitory effect of Ca^2+^, Co^2+^, Zn^2+^ and Mn^2+^ were obvious.

**Table 2:** Kinetic parameters for the wild-type CotA and Q441A using AFB_1_ as substrate.

Laccase	K_m_ (mM)^a^	*k*_cat_ (s^−1^)	*k*_cat_/K_m_ (s^−1^ mM^−1^)
WT	0.131 ± 0.004	0.054 ± 0.008	0.412
Q441A	0.084 ± 0.002	0.060 ± 0.005	0.714

a Laccase activity was calculated at 37°C with 100 mM sodium phosphate buffer (pH 8.0). AFB_1_ was chosen as substrate. K_m_ and *k*_cat_ were determined by Michaelis-Menten equation.

**Table 3:** Effect of metal ions on activity of wild-type CotA and Q441A using AFB_1_ as substrate.

Metal ion (10 mM)	Residual activity (%)		SEM	*P*-value
	WT	Q441A		
–	100^a^	100^a^	–	–
Cu^2+^	78.31 ± 11.88^bc^	86.19 ± 6.53^ab^	9.581	0.457
Mg^2+^	88.29 ± 8.46^ab^	102.20 ± 1.64^a^	6.092	0.085
Ca^2+^	70.92 ± 7.41^bc^	76.16 ± 6.16^bc^	6.817	0.484
Co^2+^	69.89 ± 4.94^bc^	68.06 ± 12.73^c^	9.656	0.859
Zn^2+^	30.03 ± 2.30^Bd^	40.99 ± 3.13^Ad^	2.750	0.016
Mn^2+^	60.40 ± 2.73^c^	61.12 ± 2.57^c^	2.652	0.798
Na^+^	86.87 ± 1.70^Bab^	97.13 ± 0.47^Aa^	1.249	0.001
SEM	4.368	4.370		
*P*-value	<0.0001	<0.0001		

^A, B^ Different letters indicate significant different (*P* < 0.05) between WT and Q441A.

^a-d^ Different letters indicate significant different (*P* < 0.05) among various metal ions.

## 4 Discussion

The selection of mutation site is a key point in site-directed mutagenesis. Current researches on site-directed mutagenesis of laccase were mostly about the changes of amino acid residues near different copper ion centers [29]. It has long been clearly recognized that mutagenesis of amino acid residues near the active center played an important role in the enhanced activity of enzymes [30]. Nevertheless, the hydrophobic interactions of amino acid residues around the active centre are important for the thermal stability of enzymes, so that the introduction of mutations around the active centre in order to improve the catalytic efficiency was possible to reduce the thermal stability of the enzymes [31]. Considering the poor thermal stability that may result from the mutation of amino acid around the active center of enzymes, we avoided the introduction of mutations near the active site, but concentrated on residues at the entrance to the substrate-binding pocket.

**Fig. 5:**
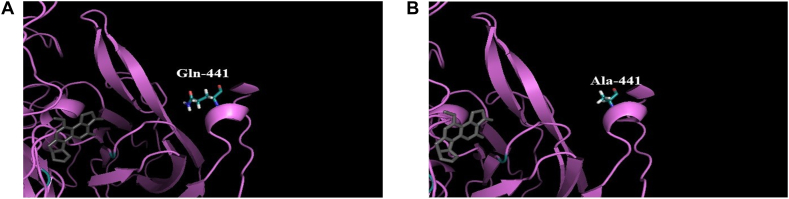
Partial structure of wild-type CotA-laccase **(A)** and Q441A mutant **(B)**. Wild-type CotA-laccase and Q441A are generated using PyMol viewer. Residue 441 is labeled and shown as colored stick, and AFB_1_ is displayed as grey stick.

**Fig. 6:**
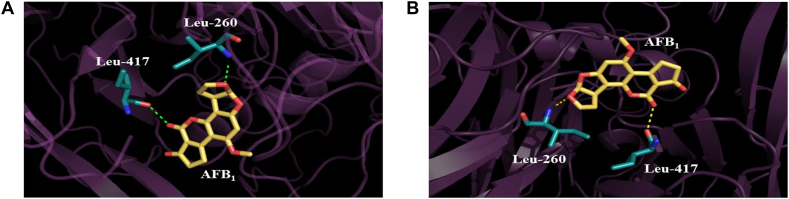
Interactions of AFB_1_ with the wild-type CotA-laccase **(A)** and Q441A **(B)**. The yellow structure indicates AFB_1_, and the green structure represents the amino acid residues around AFB_1_. Hydrogen bonds are shown as the green or yellow dashes.

In our present study, a combination of structure-based methods and site-directed mutagenesis was used to improve the detoxification ability of CotA-laccase on AFB_1_. It was found that Gln441 was located in a short α-helix fragment of the entrance of substrate-binding pocket. It had been mentioned that the change of amino acids located at the entrance of the substrate-binding pocket may reduce the steric hindrance, so that the substrate was easy to enter the tunnel and dock into the active site of the enzyme [27]. The local structures of the wild-type CotA-laccase and Q441A were further compared (Fig. 5). Both Gln441 and Ala441 could form a hydrogen bond with Ile437 according to the structural characteristics of the α-helix. However, it was apparent that the side chain of Ala441 became shorter than that of Gln441 after site-directed mutagenesis. It could be speculated that maybe Gln441 had more steric effects with the substrates than Ala441. The shorter side chain of Ala441 in the mutant contributed to the decreased steric effects, so that the substrates were more likely to bind to the active site of CotA-laccase (Fig. 5). Beyond that, the result of molecular docking showed that the mutation of Gln441 had no significant effect on the number and location of hydrogen bonds formed between AFB_1_ and the active center of CotA-laccase, so that the enzyme would not be inactivated by this kind of mutagenesis (Fig. 6).

The result showed that compared with wild-type CotA-laccase, the K_m_ value of Q441A to ABTS and AFB_1_ decreased by 1.52-fold and 1.56-fold, respectively, which indicated that the affinity of Q441A to substrates including ABTS and AFB_1_ was improved. The *k*_cat_/K_m_ value of Q441A to ABTS and AFB_1_ were determined to be 392.07 and 0.714 s^−1^ mM^−1^ which achieved 2.02 and 1.73-fold higher catalytic efficiency than that of the wild-type, respectively. Hence, it was speculated that amino acid at 441 was in an important position. When Gln441 was mutated to Ala441, the side chain became shorter and the steric hindrance decreased. Therefore, it was easy to allow the substrate to dock into the active site. The higher catalytic efficiency of Q441A for ABTS in our study was in agreement with the previous study, where Gln442 of *B. pumilus* CotA-laccase was turned into Ala442 and exhibited higher catalytic efficiency on ABTS [27]. In another research, remodeling of the substrate-binding site in the small laccase created a cavity and improved the access of substrates to the type-1 Cu site [32].

The optimal pH value of the mutant Q441A for ABTS was about 4.5, which was same to the wild-type CotA laccase. This optimal pH value was not significantly different from the optimal pH 4.0 of the *Streptomyces griseoflavus* Ac-993 laccase [33] and pH 4.4 of the *B. subtilis* LS03 laccase [34]. The wild-type CotA-laccase had the optimal reaction temperature at 80°C with ABTS as substrate, while the mutant Q441A held the optimal temperature at 90°C. Both of the optimal temperatures of wild-type CotA-laccase and Q441A to oxidize ABTS were higher than that of Lac2 from *Pleurotus pulmonarius* with 55°C [17], which indicated that maybe the optimal temperature of bacterial laccase for oxidation ABTS was higher than that of fungal laccase.

This study showed that the oxidation of AFB_1_ by CotA-laccase was enhanced remarkably after site-directed mutagenesis in a large range of pH and temperature conditions. The feed enzymes are able to used as feed additives, or they can be applied in feed processing to detoxify mycotoxins in feed directly. However, the use of enzymes to degrade mycotoxins in large-scale feed processing is time-consuming and expensive. Therefore, most of the feed enzymes on the market are used as feed additives to play a role in gastrointestinal tract of animals. As is known, the normal pH value of gastrointestinal tract of livestock is almost between 3.0 and 7.0 [[35], [36], [37]]. Q441A showed better catalytic activity than wild-type CotA-laccase using AFB_1_ as substrate under acidic or neutral conditions, especially at pH 7.0, showing that this mutant could play a more important role in the degradation of AFB_1_ in the gastrointestinal tract of animals. Since the CotA-laccase from *B. licheniformis* is a thermo-alkali stable enzyme, the stability of the wild-type CotA-laccase and Q441A were investigated. The mutant CotA-laccase was more stable at pH 9.0, therefore, Q441A had better ability to resistant strongly alkaline environments.

The degradation ratio of both wild-type CotA-laccase and Q441A to AFB_1_ was the highest at 70°C. The similar phenomenon was observed in the CotA-laccase from *B. licheniformis* ZOM-1 [38]. In addition, the optimal temperature of CotA-laccases in our study and that of CotA-laccase from *B. licheniformis* ZOM-1 to degrade AFB_1_ were higher than that of fungal laccases [21]. AFB_1_ degradability improved with the increasing temperature from 30 to 70°C was possibly related to the activation of the laccase molecules and the enhancement of the coordination of copper ions [39]. Q441A showed a relatively higher thermostability than CotA-laccase and could meet the technological requirements of feed processing [40]. Gln441 was located on the surface of CotA-laccase. The exposed Gln was prone to deamination at high temperature and not conducive to protein stability [41]. It was reported that the molecule surface hydrophobicity of mutant laccase was enhanced when Gln441 turned into Ala441 so that it would be more suitable for high temperature catalytic reaction [27].

It is of great significance to study the influence of metal ions on laccase because there are many kinds of metal ions in the environment. In this study, Zn^2+^ significantly inhibited the activity of laccase. The phenomenon was also observed in other kinds of laccases [8,42]. This was perhaps due to the interaction between metal ions and laccase electron transport system [42]. Moreover, the residual activity of laccase was over 60 % with other metal ions (Cu^2+^, Mg^2+^, Ca^2+^, Co^2+^, Mn^2+^, Na^+^) supplementation. The interesting thing was that the activity of Q441A was higher than the wild-type laccase significantly by the addition of Zn^2+^ and Na^+^. This indicated that this mutant could play a good role in the presence of heavy metals.

We obtained a CotA-laccase mutant Q441A with improved AFB_1_ detoxification in this study. However, this study has potential limitations. It is uncertain whether CotA laccase can degrade other nutrients in gastrointestinal tract of animals when used as a feed additive, and whether CotA laccase can maintain the original activity in the presence of various digestive enzymes such as trypsin and pepsin in the gastrointestinal, because these digestive enzymes may degrade CotA laccase. In the future, we plan to evaluate the application value of CotA laccase by animal feeding experiments.

## 5 Conclusion

In conclusion, this study obtained a CotA-laccase mutant Q441A with improved degradability of AFB_1_. Q441A mutant present good pH stability, thermostability and high catalytic efficiency to AFB_1_. Further studies are needed to evaluate the performance of Q441A in degrading AFB_1_ as feed additive.

## Ethics statement

All procedures performed in studies involving human participants were in accordance with the ethical standards of the institutional and/or national research committee and with the 1964 Helsinki declaration and its later amendments of comparable ethical standards. The purchase, storage and use of poisonous and harmful materials in this experiment were performed in compliance with the relevant laws and institutional guidelines of China. This article does not contain any studies with animals performed by any of the authors.

## Data availability statement

The data associated with our study have not been deposited in a publicly available repository. Data will be made available on request.

## CRediT authorship contribution statement

**Yanrong Liu:** Writing – original draft, Methodology, Investigation, Data curation. **Yongpeng Guo:** Methodology, Data curation. **Limeng Liu:** Methodology, Formal analysis. **Yu Tang:** Writing – review & editing, Formal analysis. **Yanan Wang:** Formal analysis. **Qiugang Ma:** Supervision. **Lihong Zhao:** Writing – review & editing, Supervision, Funding acquisition.

## Declaration of competing interest

The authors declare that they have no known competing financial interests or personal relationships that could have appeared to influence the work reported in this paper.

## Figures and Tables

**figs1:**
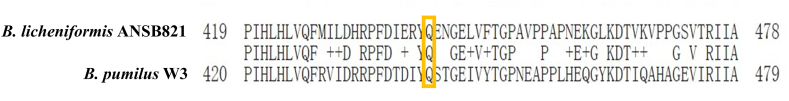


**figs2:**