# Supplementary material for: Improvement of aflatoxin B1 degradation ability by Bacillus licheniformis CotA-laccase Q441A mutant
Source: Heliyon. 2023 Nov 13;9(11):e22388. doi: 10.1016/j.heliyon.2023.e22388 (PMC10696099; doi:10.1016/j.heliyon.2023.e22388)
Supplement: Multimedia component 1 [file mmc1.docx]

**Supplemental Materials**

**Table S1.** Primer sequences

| Gene name | Direction | Nucleotide sequences (5’-3’)^a^ |
| --- | --- | --- |
| Q441A | Forward | 5’-GATATCGAGCGGTAT**GC**GGAAAACGGAGAA-3’ |
| Q441A | Reverse | 5’-**GC**ATACCGCTCGATATCAAACGGCCGGTGG-3’ |

^a^ Bold and underlined letters were mutant sites and cloning sites.

The underlined sequences indicate restriction enzyme recognition sites.

**Figure S1.** Alignment with the CotA-laccase sequences of *B. licheniformis* ANSB821 and *B. pumilus* W3 (CCTCC No. M2015018).

**Figure S2.** SDS-PAGE of purified CotA-laccase. Lane M is protein marker; lane 1 and lane 2 show the purified wild-type CotA-laccase and Q441A, respectively. The molecular weight of CotA-laccase is ~60 kDa.
